# ﻿Revision of the *javanicus* species group of the millipede genus *Glyphiulus* Gervais, 1847, with descriptions of five new species from China (Diplopoda, Spirostreptida, Cambalopsidae)

**DOI:** 10.3897/zookeys.1108.85156

**Published:** 2022-06-23

**Authors:** Yi Zhao, Wan-Ru Guo, Sergei I. Golovatch, Wei-Xin Liu

**Affiliations:** 1 College of Plant Protection, South China Agricultural University, Guangzhou 510642, China South China Agricultural University Guangzhou China; 2 Institute for Problems of Ecology and Evolution, Russian Academy of Sciences, Leninsky pr. 33, Moscow 119071, Russia Institute for Problems of Ecology and Evolution, Russian Academy of Sciences Moscow Russia

**Keywords:** Cave, DNA barcoding, new record, new species, phylogeny, taxonomy

## Abstract

The *javanicus*-group of *Glyphiulus* is re-assessed and its Chinese component species are presently divided between the following two newly-circumscribed species groups, i.e. the *formosus*- and the *sinensis*-group. The two can be differentiated, based on the diagnostic characters of the first pair of legs in the male. In addition, metatergal crests being complete and the carinotaxy formula on the collum being I–III+P+M are only characteristic of the *formosus*-group. A molecular phylogeny of the genus, based on DNA sequencing of four gene fragments of four genes, allows for *Glyphiulus* to be recovered as a monophyletic group, the phylogenetic relationship being ((Clade A, Clade B), Clade C). Molecular evidence is fully congruent with the morphological one. In addition, based on barcoding data, interspecific p-distances between *Glyphiulus* species amount to 11.2–24.9%, vs. 0–8.2% for intraspecific p-distances. Five new species of *Glyphiulus*, all cavernicolous, are described from China: *G.sinuatoprocessus* Zhao & Liu, **sp. nov.**, *G.conuliformis* Zhao & Liu, **sp. nov.** (both from Guangdong Province), *G.xiniudong* Zhao & Liu, **sp. nov.**, *G.scutatus* Zhao & Liu, **sp. nov.** and *G.portaliformis* Zhao & Liu, **sp. nov.** (all three from Guangxi Zhuang Autonomous Region). The known Chinese species of the *formosus*-group appear to mainly be confined to the South China region.

## ﻿Introduction

*Glyphiulus* Gervais, 1847 is the largest genus in the millipede family Cambalopsidae, currently comprising 70 species (Likhitrakarn et al. 2017; Liu and Wynne 2019; Jiang et al. 2021, 2022). They range from southern China in the north to Java and Borneo in the south and southeast, being particularly common in caves and usually very narrow in distribution, except for *G.granulatus* (Gervais, 1847) which is pantropical (Likhitrakarn et al. 2021).

The genus *Glyphiulus* has recently been reviewed and divided into two species groups, based on morphological characteristics alone, namely, the *granulatus*-group and the *javanicus*-group (Golovatch et al. 2007a, b; 2011a, b). The main features to distinguish these two species groups lie in the first pair of legs of the male. The *granulatus*-group is represented by a sternum with two widely separated and curved prongs, coupled with 1- or 2-segmented and strongly reduced telopodite rudiments. On the contrary, species in the *javanicus*-group show a sternum with a pair of fused, paramedian prongs, flanked by 2-segmented leg vestiges or nearly normal 4- or 5-segmented telopodites. Besides this, both groups differ in the structure of the gnathochilarium, collum and metatergal crests, as well as anterior and posterior gonopods, but these distinctions are not too stable.

There are presently 103 gene sequences related to Cambalopsidae species that can be found in NCBI. COI and 28S gene fragments used in studies on the genera *Trachyjulus* Peters, 1864, *Glyphiulus* and *Plusioglyphiulus* Silvestri, 1923 have demonstrated the genus *Trachyjulus* to be monophyletic (Likhitrakarn et al. 2020). Jiang et al. (2020, 2021) differentiated some species between *Hypocambala* Silvestri, 1895 and *Glyphiulus*, based on four gene fragments.

China currently supports the largest number of *Glyphiulus* species in the world, with 46 known species which are mainly distributed in South China’s karsts (Golovatch and Liu 2020; Jiang et al. 2022). Of these, 25 species are considered to belong to the *granulatus*-group, vs. about 21 in the *javanicus*-group.

After many years of investigation and sampling across southern China, the authors of the present paper have found out that the Chinese species from the *javanicus*-group could further be subdivided into two reliable groups, namely, the *formosus*- and the *sinensis*-group, based both on morphological and molecular evidence. However, the phylogenetic relationship between the *formosus*-, *granulatus*- and *sinensis*-group seems to be unstable. To substantiate the above new information, the present paper puts on record not only five new species, but it also adds new records for two previously-described species of *Glyphiulus* from caves in southern China. Besides this, a key to all 14 species of the *formosus*-group of *Glyphiulus* known from China is given and their distributions are mapped.

## ﻿Materials and methods

The material underlying the present study was collected by hand from several caves in southern China and preserved in 95% ethanol. The holotypes and most of the paratypes are deposited in the Zoological Collection of the South China Agricultural University (SCAU), Guangzhou, Guangdong Province, China, with a few paratypes shared with the Zoological Research Museum Koenig (ZFMK), Bonn, Germany. A detailed examination of characters and dissections were performed using a Leica S8 APO stereomicroscope. For scanning electron microscopy (SEM), the samples were cleaned by ethanol and then mounted on aluminium columns. Except for the first new species described in this paper, which was sputter-coated with gold in a Cressington 108 automatic sputter coater, the remaining four new species samples were not coated. SEM micrographs were taken using a ZEISS Sigma 300VP scanning electron microscope (based at ZFMK) or Hitachi TM4000 scanning electron microscope (based at Gongbei Port, Zhuhai City, Guangdong Province, China). After the study, dry SEM material was removed from stubs and returned to alcohol. Line drawings were prepared with a ZEISS Axioskop40 microscope with a camera lucida attached. Photographs of specimens were taken with a Keyence VHX-5000 digital microscope and edited using Adobe Photoshop CS6 software. The terminology used in the text is after Golovatch et al. (2007a, b, 2011a, b), Liu and Wynne (2019), Jiang et al. (2017, 2018, 2020) and Likhitrakarn et al. (2017, 2021). The distribution map was created using QGIS 3.20.1 software.

Genomic DNA was extracted from legs and collum tissue of specimen samples with Qiagen DNeasy Blood and Tissue kit following the manufacturer’s extraction protocol. Partial sequences of two mitochondrial genes (COI and 16S) and two nuclear genes (18S and 28S) were amplified and sequenced. The PCR amplification was performed using a T100 thermal cycler (BIO-RAD) with a final reaction volume of 25 μl. Raw sequences were edited and assembled using SeqMan Pro software (Lasergene v. 7.1; DNA Star, Inc., Madison, Wis., USA).

Protein-coding gene sequences (COI) were aligned using the codon-aware programme MACSE v. 2.03 (Ranwez et al. 2018), which preserves reading frame and allows incorporation of sequencing errors or sequences with frameshifts. The more variable sequences (16S, 18S, 28S) were aligned using the online version of MAFFT v. 7.0 (Katoh and Standley 2013) using ‘—auto’ strategy and normal alignment mode. Best partitioning scheme and evolutionary models for six pre-defined partitions were selected using PartitionFinder2 (Lanfear et al. 2017), with all algorithm and AICc criteria.

The analysis involved 37 *Glyphiulus*, two *Plusioglyphiulus* and five *Trachyjulus* COI sequences (18 new sequences and 26 from GenBank). Codon positions included were 1^st^+2^nd^+3^rd^. All positions containing ‘N’s were removed for each sequence pair. Uncorrected p-distances of COI markers were calculated using MEGA X (Kumar et al. 2018).

The final aligned dataset included 28 sequences, each with 657 bp of COI (one is not available for SCAUG32), 481 bp of 16S rRNA, 627 bp of 18S rRNA and 1182 bp of 28S rRNA. The combined analysis after these exclusions consisted of 2947 positions. Maximum Likelihood (ML) and Bayesian Inference (BI) analyses were executed by PhyloSuite v.1.2.2 (Zhang et al. 2020). ML analysis was conducted using IQ-TREE with 1000 bootstrap replications. Bayesian Inference (BI) analysis was implemented by MrBayes 3.2.6 using the Markov Chain Monte Carlo technique (MCMC) (Ronquist et al. 2012) under partition model (two parallel runs, 2,000,000 generations), in which the initial 25% of sampled data were discarded as burn-in.

All analysed species, voucher numbers/taxonomy ID, and Genbank accession numbers are listed in Table 1.

**Table 1.** List of the species used for molecular phylogenetic analyses and their relevant information. *: new sequence; /: absent.

Voucher number	Species	GenBank accession numbers			
		COI	16S	18S	28S
SCAUG33*	*Glyphiulus* sp. 1	ON255879	ON617345	ON263093	ON263226
SCAUWL49*	*Glyphiulus* sp. 1	ON255892	ON617360	ON263096	ON263229
SCAUWL20*	*Glyphiulus* sp. 2	ON256155	ON617353	ON263101	ON263239
SCAUG32*	*Glyphiuluszorzini*	/	ON263092	ON263225	ON263092
SCAUWL23*	*Glyphiuluszorzini*	ON255887	ON263095	ON263228	ON263095
SCAUG39*	*Glyphiulus* sp. 3	ON255880	ON263094	ON263227	ON263094
IBGASJXK051	*Glyphiulusguangnanensis*	MN725096	MN733292	MN733302	MN733282
SCAUWL38*	*Glyphiulusimpletus*	ON255889	ON617357	ON263088	ON263235
SCAUWL39*	*Glyphiulusimpletus*	ON255890	ON617358	ON263090	ON263236
IBGASJXK002	*Glyphiulusimpletus*	MN725095	MN733291	MN733301	MN733281
SCAUG78*	*Glyphiulusxiniudong* Zhao & Liu, sp. nov.	ON255885	ON617351	ON263085	ON263232
SCAUWL37*	*Glyphiuluscalceus*	ON255888	ON617356	ON263089	ON263234
IBGASJXK061	*Glyphiuluscalceus*	MN725098	MN733294	MN733304	MN733284
SCAUG72*	*Glyphiulusscutatus* Zhao & Liu, sp. nov.	ON255884	ON617350	ON263084	ON263231
SCAUWL30*	*Glyphiulusscutatus* Zhao & Liu, sp. nov.	ON256153	ON617355	ON263087	ON263238
IBGASJXK059	*Glyphiulusfoetidus*	MN725097	MN733293	MN733303	MN733283
SCAUWL40*	*Glyphiulusportaliformis* Zhao & Liu, sp. nov.	ON255891	ON617359	ON263091	ON263237
JXK275	*Glyphiulussinuatoprocessus* Zhao & Liu, sp. nov.	OM746179	/	/	/
SCAUWL02*	*Glyphiulussinuatoprocessus* Zhao & Liu, sp. nov.	ON255886	ON617352	ON263086	ON263233
SCAUG24*	*Glyphiulusconuliformis* Zhao & Liu, sp. nov.	ON255878	ON617343	ON263083	ON263230
SCAUG15*	*Glyphiulusdeharvengi*	ON255877	ON617342	ON263097	ON263221
IBGASJXK310	*Glyphiulusdeharvengi*	MN725104	MN733300	MN733310	MN733290
IBGASJXK072	*Glyphiulusquadrohamatus*	MN725099	MN733295	MN733305	MN733285
IBGASJXK196	*Glyphiulusgranulatus*	MN725102	MN733298	MN733308	MN733288
SCAUG50*	*Glyphiulusproximus*	ON255881	ON617347	ON263098	ON263222
SCAUG61*	*Glyphiulusproximus*	ON255882	ON617348	ON263099	ON263223
SCAUG62*	*Glyphiulusspeobius*	ON255883	ON617349	ON263100	ON263224
CAM022	*Glyphiulusduangdee*	MN893779	/	/	/
CAM030	*Glyphiulussattaa*	MN893778	/	/	/
JXK282	*Glyphiulusformosus*	MN905180	/	/	/
JXK375	*Glyphiulusfortis*	OM746180	/	/	/
JXK376	*Glyphiulusfortis*	OM746181	/	/	/
JXK377	*Glyphiulusfortis*	OM746182	/	/	/
XK046	*Glyphiulushainanensis*	OM746174	/	/	/
XK047	*Glyphiulushainanensis*	OM746175	/	/	/
XK048	*Glyphiulushainanensis*	OM746176	/	/	/
XK049	*Glyphiulushainanensis*	OM746177	/	/	/
XK050	*Glyphiulushainanensis*	OM746178	/	/	/
IBGAS JXK517	*Cambalaannulata*	MT683305	MT676457	MT676456	MT676769
IBGAS JXK165	*Hypocambalazizhongi*	MN725101	MN733297	MN733307	MN733287
CAM031	*Plusioglyphiuluserawan*	MN893780	/	/	/
CAM021	*Plusioglyphiulussaksit*	MN893781	/	/	/
CAM059	*Trachyjulusbifidus*	MN893771	/	/	/
CAM061	*Trachyjulusbifidus*	MN893772	/	/	/
CAM027	*Trachyjulusphylloides*	MN893773	/	/	/
CAM079	*Trachyjulusunciger*	MN893774	/	/	/
CAM070	*Trachyjulusmagnus*	MN893775	/	/	/

## ﻿Results

### ﻿Morphologic analysis

**Figure 1.**
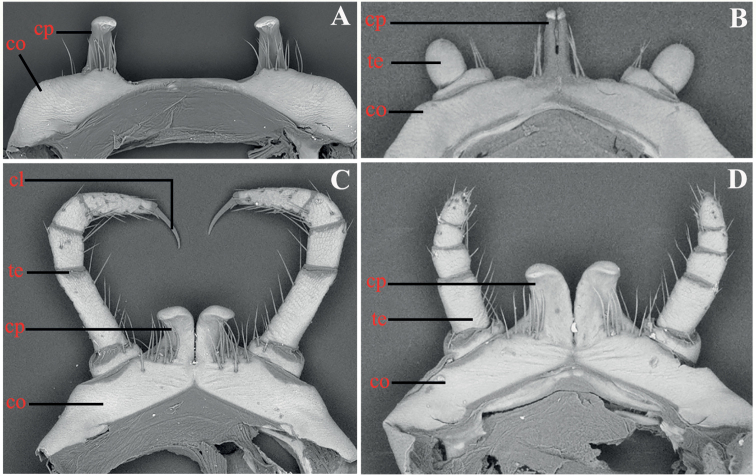
♂ leg 1 of *Glyphiulus*, frontal view **A***granulatus*-group **B***formosus*-group **C, D***sinensis*-group. Abbreviations: cl: claw, co: coxosternum, cp: coxosternum process, te: telopodite.

The Chinese *Glyphiulus* species could be divided into three morphologically distinct species groups, mainly based on the structure of male legs 1 (Fig. 1): the *granulatus*-group (Fig. 1A, represented by a coxosternum bearing strongly separated, distally evidently curved prongs, each side with or without a rather small leg vestige), the *formosus*-group (Fig. 1B, showing a pair of small, fused, paramedian, subunciform prongs directed forward, flanked by strongly separated, rudimentary, 2-segmented leg vestiges) and the *sinensis*-group (Fig. 1C, D, telopodites often complete or nearly so, with or without claw; coxosternum with a pair of relatively large and stout, paramedian, basically non-fused outgrowths directed laterad).

**Figure 2.**
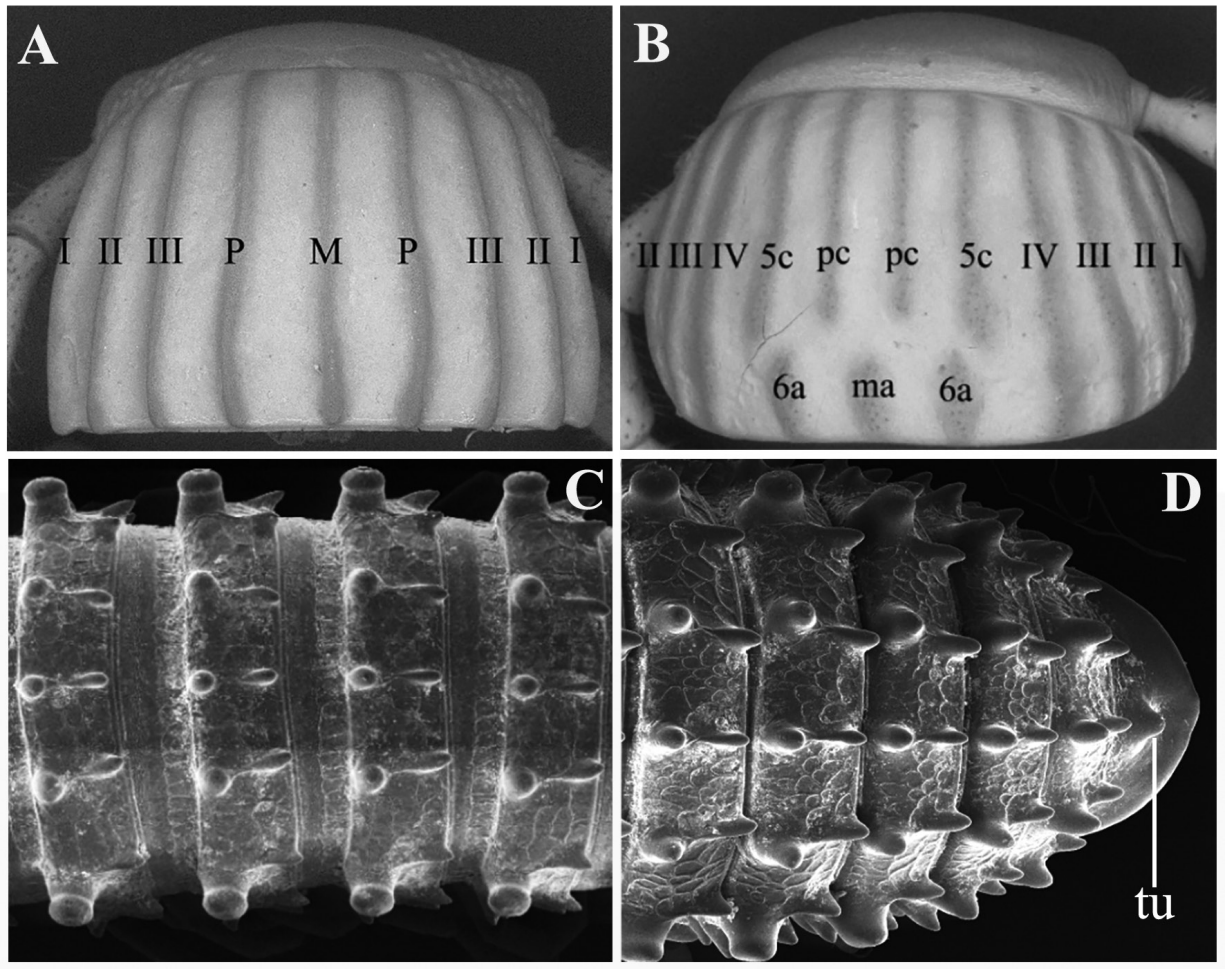
Partial trunk morphology of *Glyphiulus***A** collum’s carinotaxy formula I–III+P+M **B** collum’s carinotaxy formula I–IV+5c+6a+pc+ma **C** metaterga carinotaxy formula 2/2+I/i+3/3, dorsal view **D** epiproct with a strong central tubercle dorsally. Abbreviation: tu: tubercle.

Species of the *formosus*-group are also distinguished by the following characteristics (Fig. 2): (1) Rather complete crests on collum, carinotaxy formula I–III+P+M (Fig. 2A), vs. crests incomplete, carinotaxy formula either I–IV+5c+6a+pc+ma (Fig. 2B) or something similar. (2) Metatergal carinotaxy formula 2/2+I/i+3/3 (Fig. 2C) or nearly so, but never 2/2+I/i+4/3. (3) Epiproct basically with a strong central tubercle dorsally (Fig. 2D). (4) Anterior gonopod with a scalloped shield-shaped coxosternum or coxite with an apicomesal process and posterior gonopods always with flagella.

### ﻿Distance analysis

The number of base differences per site between sequences (absolute p-distance) is shown in Suppl. material 1: Table S1. Uncorrected pairwise distances between *Glyphiulus* and the other two genera of cambalopsid species were found to be generally high, varying 17.7–27.1% between *Glyphiulus* and *Trachyjulus* and 17.2–24.7% between *Glyphiulus* and *Plusioglyphiulus*.

Amongst the *Glyphiulus* species concerned, *G.foetidus* showed the highest divergence from the other *Glyphiulus* species, ranging from 14.4–24.9%. The lowest divergence was 11.2% between *Glyphiulus* sp.1 SCAUG33, SCAUWL49 and *G. Glyphiulus* sp. 2 SCAUWL20.

The intraspecific divergence of *Glyphiulus* species was found to range from 0.0–8.2%. Intraspecific distances in our dataset between individuals of *G.impletus* vary 2.4–8.2%, 2.1% in *G.calceus*, 0.0–3.3% in *G.hainanensis* and 6.6% in *G.scutatus* Zhao & Liu, sp. nov.

### ﻿Phylogenetic analysis

**Figure 3.**
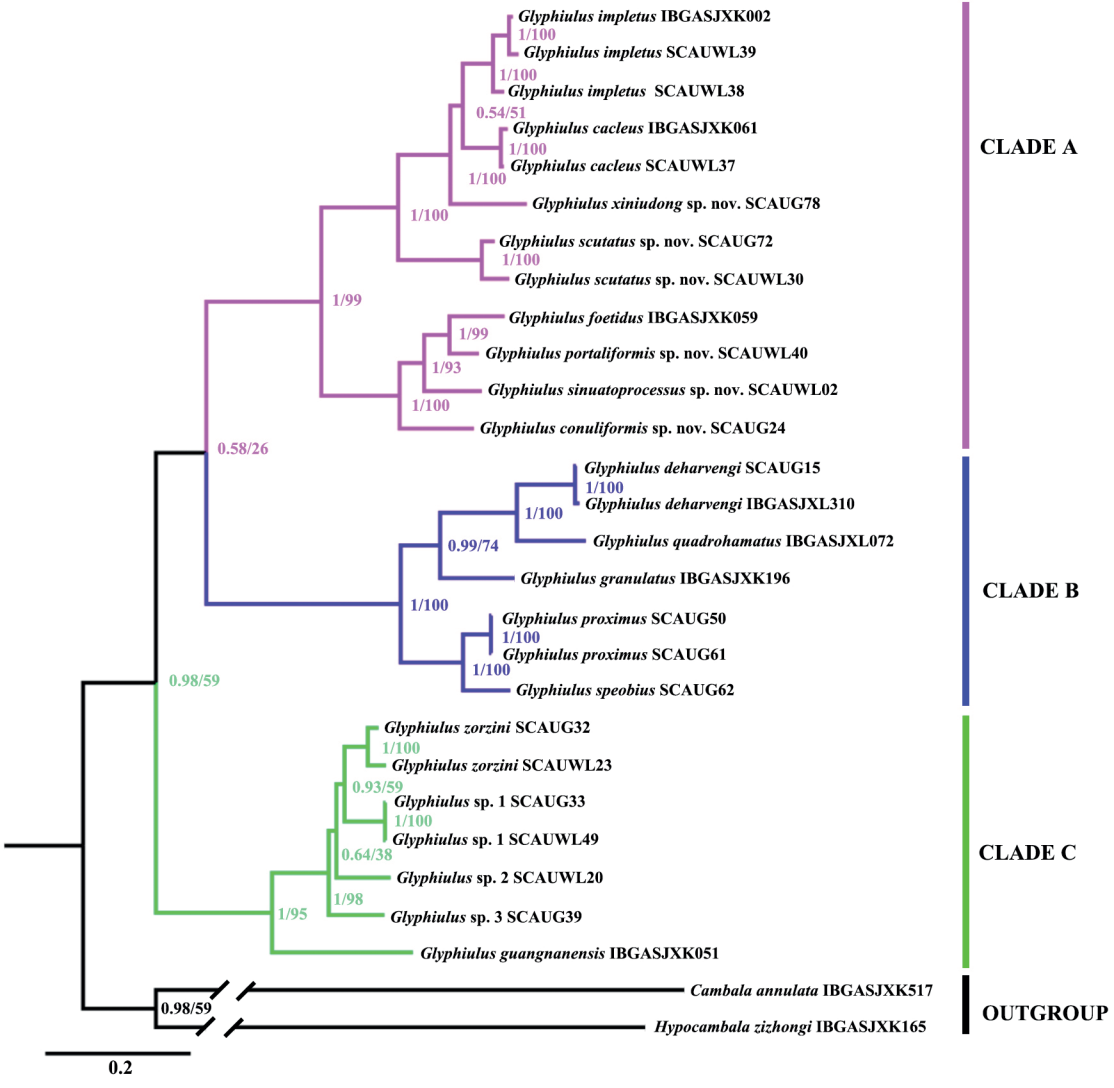
Phylogenetic reconstruction of the genus *Glyphiulus* species, based on four gene fragments. Numbers on nodes indicate Bayesian posterior probability (bpp) from Bayesian Inference analysis (BI) and bootstrap values from Maximum Likelihood (ML).

As the phylogenetic tree estimated by both the Maximum Likelihood (ML) and Bayesian Inference (BI) analyses revealed equivalent topologies, we only present the BI tree here (Fig. 3). The monophyly of the genus *Glyphiulus* was strongly supported by 0.98 bpp for BI, but a little weaker at 59% bootstrap values for ML. The analysed species of *Glyphiulus* can be clearly divided into three clades, the phylogenetic relationships amongst them being ((Clade A, Clade B), Clade C). The three clades can be defined as three species groups, namely, the *formosus*-group (Clade A), the *granulatus*-group (Clade B) and the *sinensis*-group (Clade C). The former two clades are sister-groups with 0.58 bpp for BI and a 26% bootstrap support.

Within Clade A, almost all internal nodes were strongly supported: 0.54–1 bpp for BI and 51–100% bootstrap values for ML. *Glyphiulusfoetidus* and three new species (*G.portaliformis* Zhao & Liu, sp. nov., *G.sinuatoprocessus* Zhao & Liu, sp. nov. and *G.conuliformis* Zhao & Liu, sp. nov.) found their places in the basal part of the tree, followed by *G.scutatus* Zhao & Liu, sp. nov. and a sister clade of *G.xiniudong* Zhao & Liu, sp. nov., *G.calceus* and *G.impletus* (Fig. 3).

**Figure 4.**
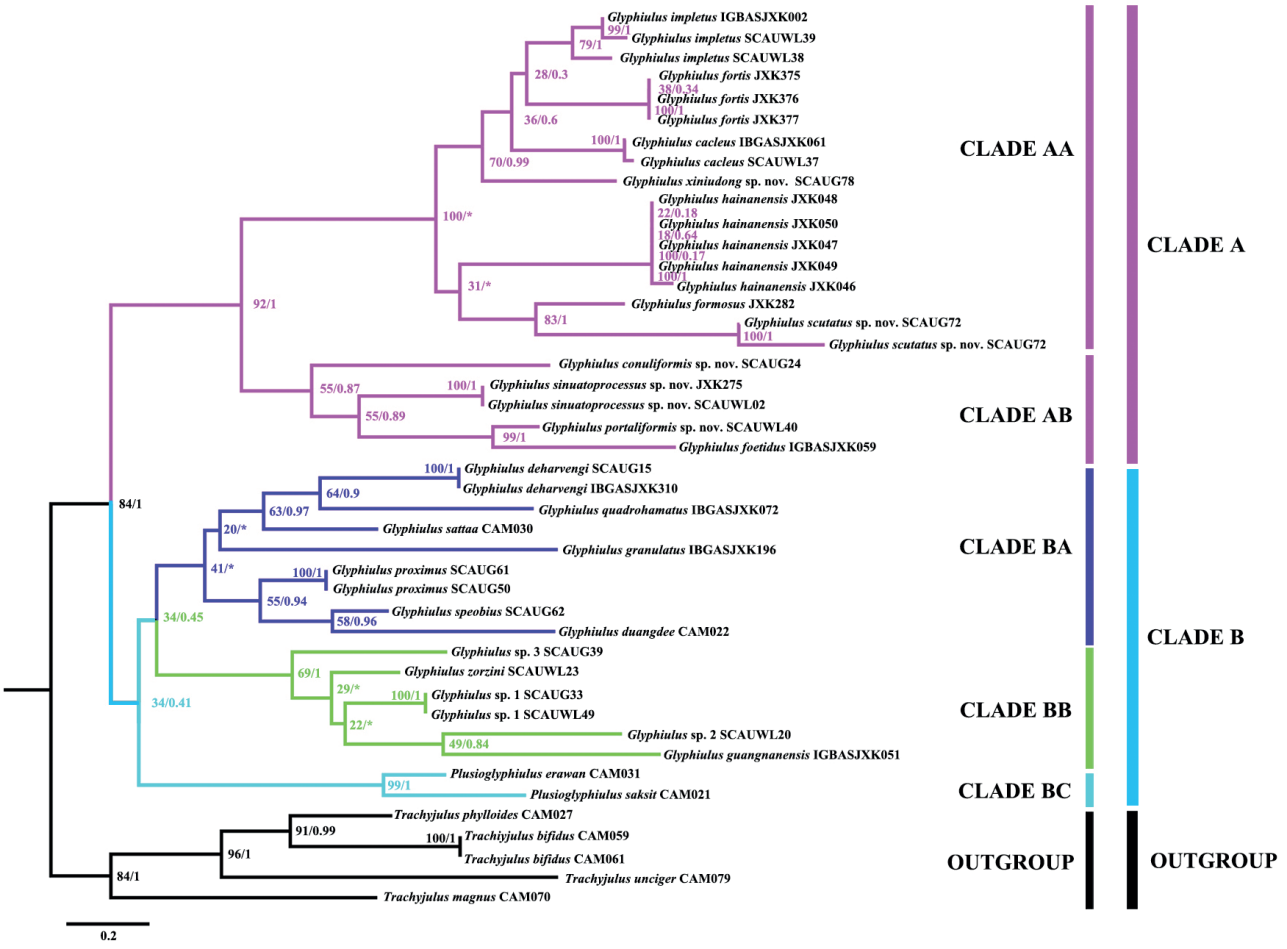
Phylogenetic tree of the mitochondrial COI gene for *Glyphiulus* constructed using Maximum Likelihood analysis. Numbers on branches are estimates of the bootstrap values and bpp of a clade, respectively. *: inconsistent construction of ML and BI.

In the single gene (COI) tree from the ML analysis, *Trachyjulus* species served as an outgroup and were clearly prioritised (Fig. 4). However, *Plusioglyphiulus* (Clade BC) became the sister clade of the ingroup which, together with the *granulatus*-group (Clade BA) and the *sinensis*-group (Clade BB), formed Clade B. In addition, clade A (the *formosus*-group) was obviously divided into two small clades. Clade AA included *G.impletus*, *G.fortis*, *G.calceus*, *G.xiniudong* Zhao & Liu, sp. nov., *G.hainanensis*, *G.formosus* and *G.scutatus* Zhao & Liu, sp. nov., while Clade AB consisted of *G.conuliformis* Zhao & Liu, sp. nov., *G.sinuatoprocessus* Zhao & Liu, sp. nov., *G.portaliformis* Zhao & Liu, sp. nov. and *G.foetidus*.

Molecular evidence is thereby fully congruent with the morphological one.

### ﻿Taxonomic treatment

**Family Cambalopsidae Cook, 1895**

#### Genus *Glyphiulus* Gervais, 1847

##### ﻿ Glyphiulus sinuatoprocessus

Taxon classificationAnimaliaSpirostreptidaCambalopsidae

Zhao & Liu sp. nov.

F11308F2-4721-5427-8D73-BB43F8BBA481

http://zoobank.org/A8C259A1-4697-4890-8E87-442953B8F104

Figs 5A , 6 , 7

###### Type material.

***Holotype*** ♂ (SCAU GD7), China, Guangdong Province, Qingyuan City, Yangshan County, Taiping Town, Niubi Village, Cave Niubi Yan, 24°10'23.93"N, 112°33'27.50"E, 100 m alt., 2014-XII-27, leg. Tian Mingyi, Liu Weixin, Huang Sunbin & Wang Xinhui. ***Paratypes***: 1 ♂ (ZFMK), 1 ♂, 1 ♀ (SEM), 2 ♂, 1 ♀ (SCAU GD7), same data as the holotype.

###### Etymology.

To emphasise the apicomesal process of the anterior gonopod coxite being hook-shaped.

###### Diagnosis.

Differs from congeners of the *formosus*-group by the anterior gonopod showing a high and digitiform process, in which the tip is hook-shaped, coupled with a short, distally pectinate flagellum of the posterior gonopod. Based on molecular evidence, *G.sinuatoprocessus* Zhao & Liu, sp. nov. differs from all other *Glyphiulus* species analysed in a > 16.1% uncorrected p-distance of the COI barcoding gene.

###### Description.

**Figure 5.**
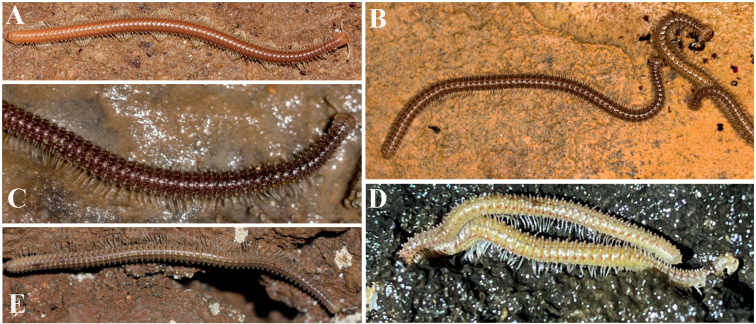
Pictures of live animals **A***G.sinuatoprocessus* Zhao & Liu, sp. nov. from Cave Niubi Yan **B***G.scutatus* Zhao & Liu, sp. nov. from Cave Bianfu Dong **C***G.portaliformis* Zhao & Liu, sp. nov. from Cave Baiyan Dong **D***G.xiniudong* Zhao & Liu, sp. nov. from Cave Xiniu Dong **E***G.conuliformis* Zhao & Liu, sp. nov. from Cave Yanzi Dong.

Length ca. 33.0–42.0 (♂) or 45.0–50.0 mm (♀), mid-body rings round in cross-section, their width and height similar, 2.2–2.3 mm (♂) or 2.5–2.8 mm (♀). Body with 52–65 (♂) or 70–76 (♀) podous + 1–4 apodous rings + telson. Colouration orange-brownish to brownish, anterior part of body much darker in alcohol, red-brownish (Fig. 5A).

***Head*** surface smooth (Fig. 7A). Labrum with 4 or 5 teeth anteromedially. Ocellaria blackish, with 12–15 (♂) or 17–23 (♀) ommatidia arranged in 1–3 irregular linear rows (Fig. 7A). Antennae relatively long, reaching back to ring 3; in length, antennomeres 5 > 3 > 2 > 4 > 6 > 1 > 7. Antennomeres 5–7 each with a distodorsal field or corolla of bacilliform sensilla (sensory bacilli). Antennomere 7 with four sensory cones (Fig. 7A). Gnathochilarium with a separate promentum, lamellae linguales and promentum densely setose, a few setae on mentum (Fig. 7B). Mandible with a large external tooth and an internal tooth, the latter provided with nine cusps.

**Figure 6.**
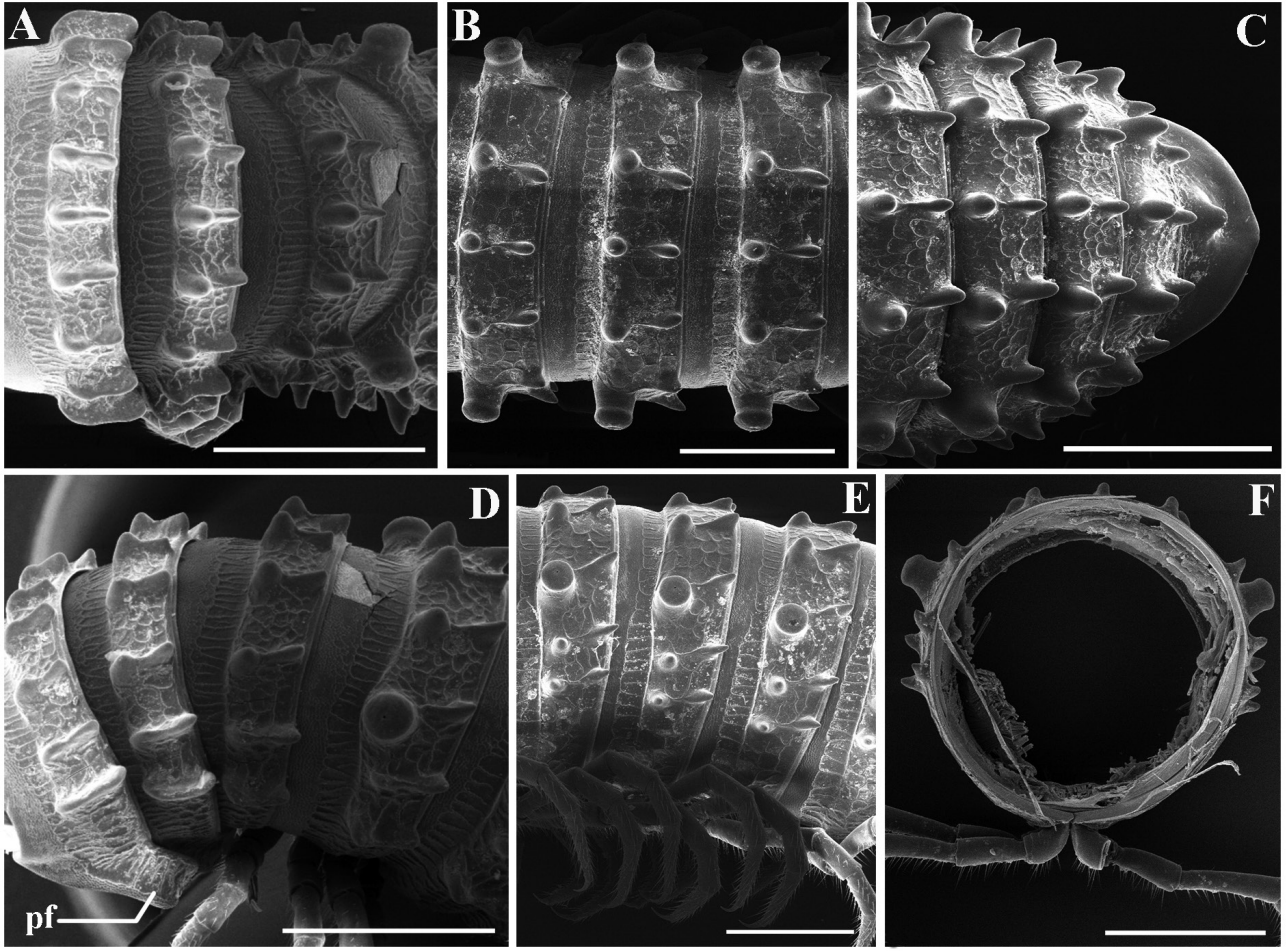
*Glyphiulussinuatoprocessus* Zhao & Liu, sp. nov., ♂ paratype **A** rings 2–4, dorsal view **B, E** mid-body rings, dorsal and lateral views, respectively **C** posterior body rings, dorsal view **D** rings 2–5, sublateral view **F** cross-section of a mid-body ring, caudal view. Scale bars: 1 mm. Abbreviation: pf: pleural flap.

**Figure 7.**
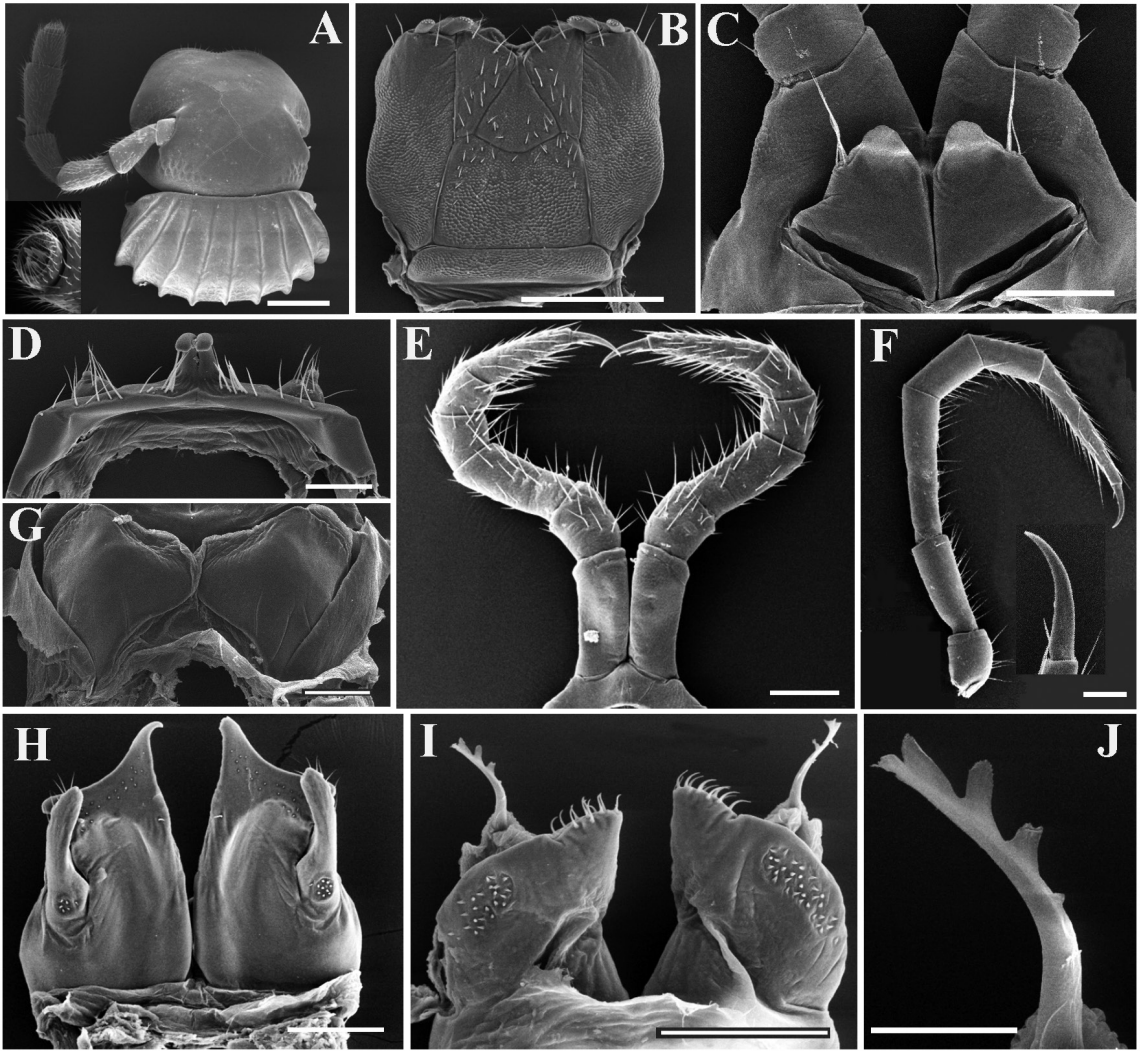
*Glyphiulussinuatoprocessus* Zhao & Liu, sp. nov., ♂ paratype **A** head, collum and antenna **B** gnathochilarium **C** penes **D** leg 1, frontal view **E** leg 3, frontal view **F** mid-leg **G** ♀ paratype, vulvae **H** anterior gonopods, caudal view **I** posterior gonopod, caudal view **J** flagellum. Scale bars: 0.5 mm (**A, B**), 0.2 mm, (**C–I**), 0.1 mm (**J**).

***Collum***: crests complete and evident; carinotaxy formula I–III+P+M (Fig. 7A). Following metaterga strongly crested; carinotaxy formula 2/2+I/i+3/3 (Fig. 6). Ozoporiferous tubercles very large, much higher than wide. Tegument delicately alveolate-areolate, fine longitudinal striations in front of stricture. Metatergal setae absent. Rings 2 and 3 with long pleural flaps (Fig. 6D). Limbus more or less regularly denticulate.

***Epiproct*** simple, very narrow, caudal edge uneven, with a strong central tubercle dorsally (Fig. 6C). Paraprocts rather regularly convex, each with several irregular rows of setae. Hypoproct transversely bean-shaped, with 3+3 small setae near caudal margin.

***Legs*** slender, about 1.2 times as long as mid-body height; claw with a small accessory spine at base, about 1/3 as long as claw (Fig. 7F). ♂ legs 1 very strongly reduced, represented by a sternum showing a pair of small, fused, paramedian, subunciform prongs directed forward, with about 10–11+10–11 long setae at base; flanked by strongly separated, rudimentary, 2-segmented leg vestiges, first segment being much larger (Fig. 7D). ♂ legs 2 slightly hypertrophied, coxae large; penes small, much shorter than coxae, oblong-subtrapeziform, each with two strong setae distolaterally (Fig. 7C). ♂ legs 3 modified through coxae being especially slender and elongate (Fig. 7E). ♂ femora 6 and 7 normal, without modifications.

***Anterior gonopods*** (Fig. 7H) with a broad and plate-shaped coxosternum supporting about 18–20 microsetae near distal margin; apicomesal process of coxite very high, digitiform, tip narrow and hook-shaped. Telopodite large and coiled, 1-segmented, lateral in position, slightly higher than lateral corner of coxite and with a field of 5–10 microspinules at base and 6–8 strong setae apically.

***Posterior gonopods*** (Fig. 7I) compact. Coxite subquadrate, with a circular field of 12–15 basolateral microspinules in frontal view and an elongated field of 20–25 microspinules in caudal view; apical and mesal parts of coxite with dense, strong and curved setae. Lamelliform lobe membranous, with a short, distally pectinate flagellum (Fig. 7J).

***Vulvae*** very simple, bare, modestly emarginate medially (Fig. 7G).

###### Remarks.

In the absence of direct troglomorphic traits, this species can only be considered as troglophilic at most. In the Guangdong Province and in Hong Kong, only *G.formosus* and *G.granulatus* have been recorded as yet.

##### ﻿ Glyphiulus conuliformis

Taxon classificationAnimaliaSpirostreptidaCambalopsidae

Zhao & Liu sp. nov.

CB523FEB-7A25-5460-9F1B-4086B3618D6B

http://zoobank.org/7FCC4E95-5E67-450E-8003-D5F41C71E5B1

Figs 5E , 8 , 9

###### Type material.

***Holotype*** ♂ (SCAU G24), China, Guangdong Province, Yangjiang City, Yangchun, Cave Yanzi Dong, 22°5'N, 111°36'50"E, 400 m alt., 2016-X-29, leg. Tian Mingyi, Chen Mengzhen & Wang Dianmei. ***Paratypes***: 2 ♂, 26 ♀ (SCAU G24), same data as the holotype.

###### Etymology.

To emphasise the metatergal anterior tubercles being very sharp and coniform.

###### Diagnosis.

Differs from congeners of the *formosus*-group by the conical shape of the anterior tubercles of metaterga and by the first segment of the telopodite being significantly enlarged in ♂ legs 1, combined with the anterior gonopod process being slender, finger-shaped and curved inwards distally. Based on molecular evidence, *G.conuliformis* Zhao & Liu, sp. nov. differs from all other *Glyphiulus* species analysed in a > 17.0% uncorrected p-distance of the COI barcoding gene.

###### Description.

Length ca. 32.5–37.0 (♂) or 47.0–58.0 mm (♀), mid-body rings round in cross-section, their width and height similar, 1.7–2.0 mm (♂) or 2.2–3.0 mm (♀). Body with 48–53 (♂) or 57–67 (♀) podous + 2–4 apodous rings + telson. Colouration dark brownish, head and legs yellowish (Fig. 5E).

***Head*** surface smooth. Labrum with four teeth anteromedially (Fig. 9C). Ocellaria blackish, with 12–22 ommatidia arranged in 2–3 irregular linear rows (Fig. 9A). Antennae slender, slightly clavate, reaching back to middle of rings 2 and 3; in length, antennomeres 5 > 3 > 2 ≈ 4 > 6 > 1 > 7. Antennomeres 5–7 each with a distodorsal field or corolla of bacilliform sensilla (sensory bacilli). Antennomere 7 with four sensory cones (Fig. 9B). Gnathochilarium with a separate promentum, polytrichous on promentum and mentum, lamellae linguales each with 6–7 setae (Fig. 9C). Mandible not dissected.

**Figure 8.**
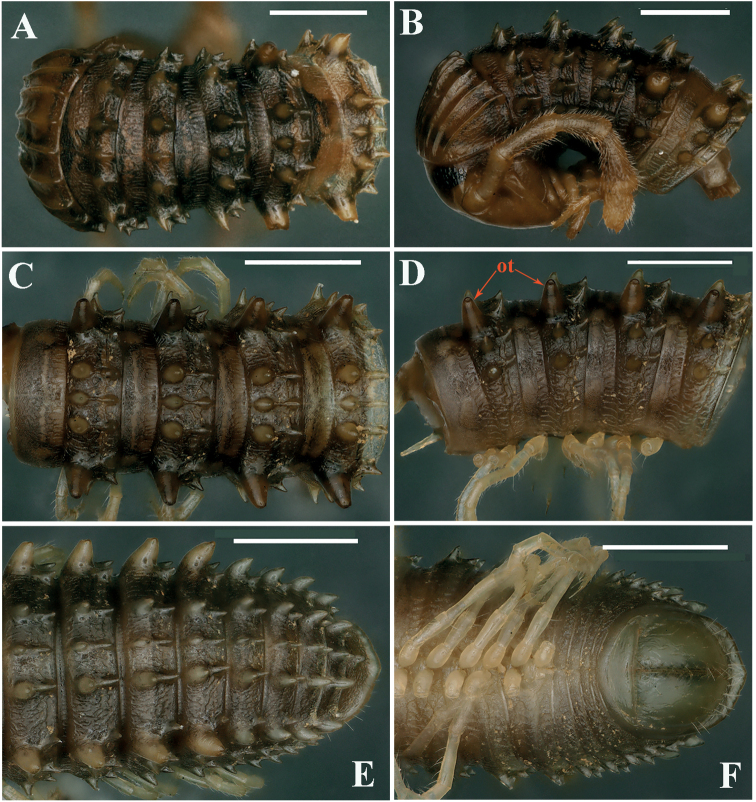
*Glyphiulusconuliformis* Zhao & Liu, sp. nov., ♂ paratype **A, B** anterior body rings, dorsal and lateral views **C, D** mid-body rings, dorsal and lateral views, respectively **E, F** posterior body rings, dorsal and ventral views, respectively. Scale bars: 1 mm. Abbreviation: ot: ozoporiferous tubercle.

***Collum***: crests complete and evident; carinotaxy formula I–III+P+M (Figs 8A, B). Following metaterga strongly crested, anterior tubercles very sharp and coniform; carinotaxy formula 2/2+I/i+3/3 (Fig. 8). Ozoporiferous tubercles very large, coniform (Figs 8C, D). Tegument delicately alveolate-areolate, fine longitudinal striations in front of stricture. Rings 2 and 3 with long pleural flaps.

***Epiproct*** simple, caudal edge with a very low central protrusion, dorsally with an obvious sharp tubercle (Fig. 8E). Paraprocts regularly convex, each with several irregular rows of setae (Fig. 8F). Hypoproct transversely bean-shaped, with 3+3 small setae.

**Figure 9.**
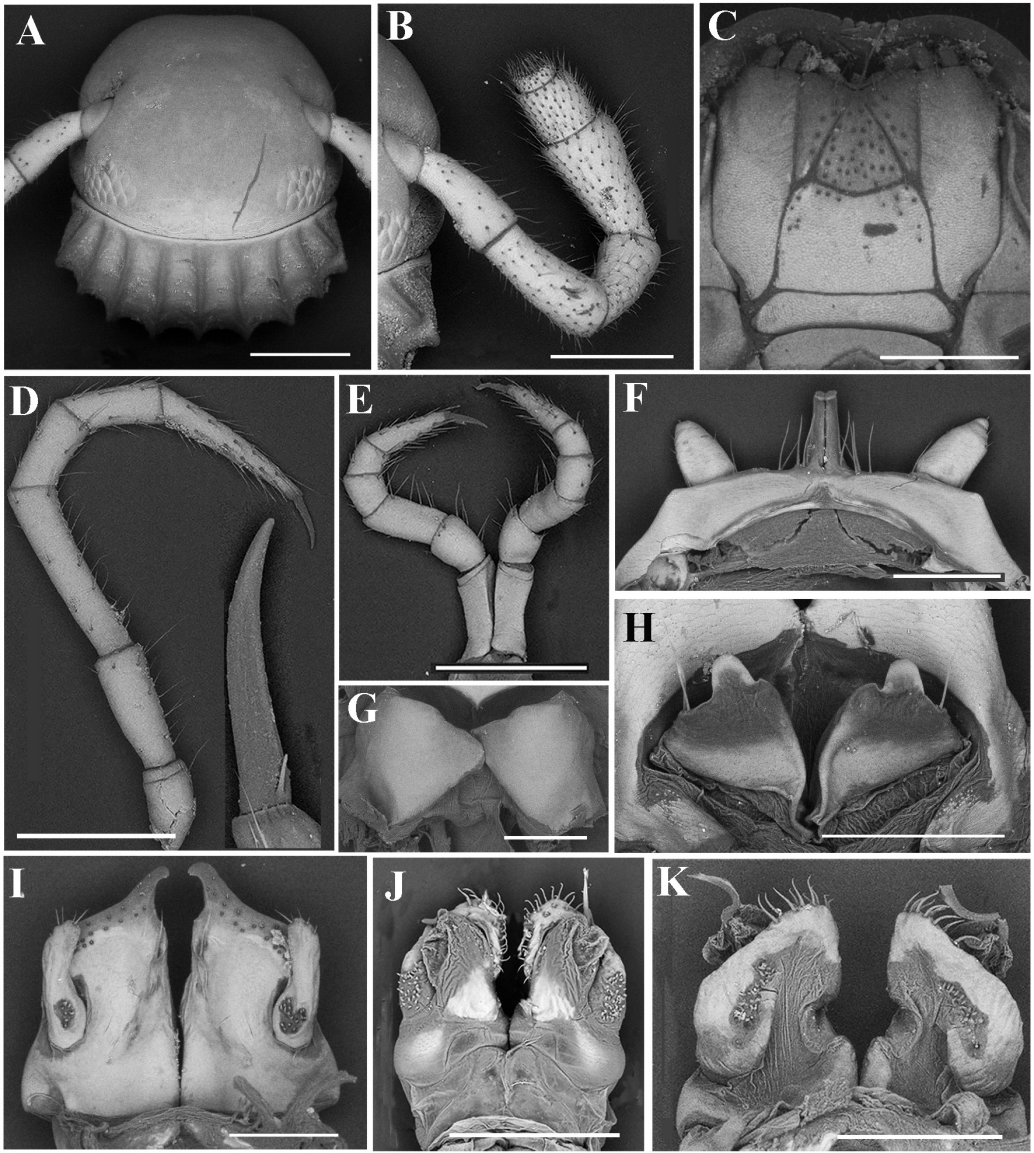
*Glyphiulusconuliformis* Zhao & Liu, sp. nov., ♂ paratype **A** head and collum **B** right antenna, oral view **C** gnathochilarium **D** mid-leg and claw **E** leg 3, caudal view **F** leg 1, frontal view **G** ♀ paratype, vulvae **H** penes **I** anterior gonopods, caudal view **J, K** posterior gonopods, frontal and caudal views, respectively. Scale bars: 0.5 mm (**A–E**), 0.2 mm (**F–K**).

***Legs*** slender, about 1.3 times as long as mid-body height; claw with a small accessory spine at base, about 1/6 as long as claw (Fig. 9D). ♂ legs 1 very strongly reduced, represented by a sternum showing a pair of small, unfused, paramedian, subunciform prongs directed forward, with about 4–5+4–5 long setae at base; flanked by strongly separated, rudimentary, 2-segmented, asymmetric leg vestiges, first segment significantly enlarged (Fig. 9F). ♂ legs 2 slightly hypertrophied, coxae large; penes rather small, much shorter than coxae, oblong-subtrapeziform, each with 1 or 2 strong setae distolaterally (Fig. 9H). ♂ legs 3 modified through coxae being especially slender and elongate (Fig. 9E). ♂ femora 6 and 7 normal, without modifications.

***Anterior gonopods*** (Fig. 9I) with a broad and plate-shaped coxosternum supplied with about 14–16 microsetae near distal margin; apicomesal process of coxite high, slender and digitiform, curved inwards distally. Telopodite very large and stout, coiled, 1-segmented, lateral in position, almost parallel to lateral corner of coxite, with a field of 8–16 microspinules at base and five strong setae apically.

***Posterior gonopods*** (Figs 9J, K) compact. Coxite subtrapezoid, with a longitudinal field of 22–24 basolateral microspinules in frontal view and a slanted field of 32–36 median microspinules in caudal view; apical and mesal parts of coxite with dense, strong and curved setae. Lamelliform lobe membranous, with a rather short and broad flagellum.

***Vulvae*** very simple, bare, M-shaped (Fig. 9G).

###### Remark.

In the absence of direct troglomorphic traits, this species can only be considered as troglophilic at most.

##### ﻿ Glyphiulus xiniudong

Taxon classificationAnimaliaSpirostreptidaCambalopsidae

Zhao & Liu sp. nov.

FCF9A2D3-31C5-523D-AE28-7198FC9D518C

http://zoobank.org/6F0C1D58-BCAB-4FF0-AA4E-22D189E7202D

Figs 5D , 10 , 11

###### Type material.

***Holotype*** ♂ (SCAU G78), China, Guangxi Zhuang Autonomous Region, Laibin City, Wuxuan County, Cave Xiniu Dong, 23°33'N, 109°32'55"E, 100 m alt., 2021-I-15, leg. Tian Mingyi, Liu Weixin & Zhao Yi. ***Paratypes***: 4 ♂, 30 ♀ (SCAU G78), same data as the holotype.

###### Etymology.

To emphasise the provenance of this species from the “Xiniu” Cave, in Chinese meaning “rhinoceros”; noun in apposition.

###### Diagnosis.

Differs from congeners of the *formosus*-group by the metazonae with an obvious, corrugate, carved texture and by the leg claw with a rather large accessory spine, coupled with a long subtriangular coxosternum of the anterior gonopod, the latter process being narrow and digitiform. Based on molecular evidence, *G.xiniudong* Zhao & Liu, sp. nov. differs from all other *Glyphiulus* species analysed in a > 14.7% uncorrected p-distance of the COI barcoding gene.

###### Description.

Length ca. 28.0–31.0 (♂) or 26.0–41.0 mm (♀), mid-body rings round in cross-section, their width and height similar, 1.1–1.3 (♂) or 1.2–1.8 mm (♀). Body with 42–54 (♂) or 45–64 (♀) podous + 0–3 apodous rings + telson. Colouration purple-brownish, legs almost transparent (Fig. 5D).

**Figure 10.**
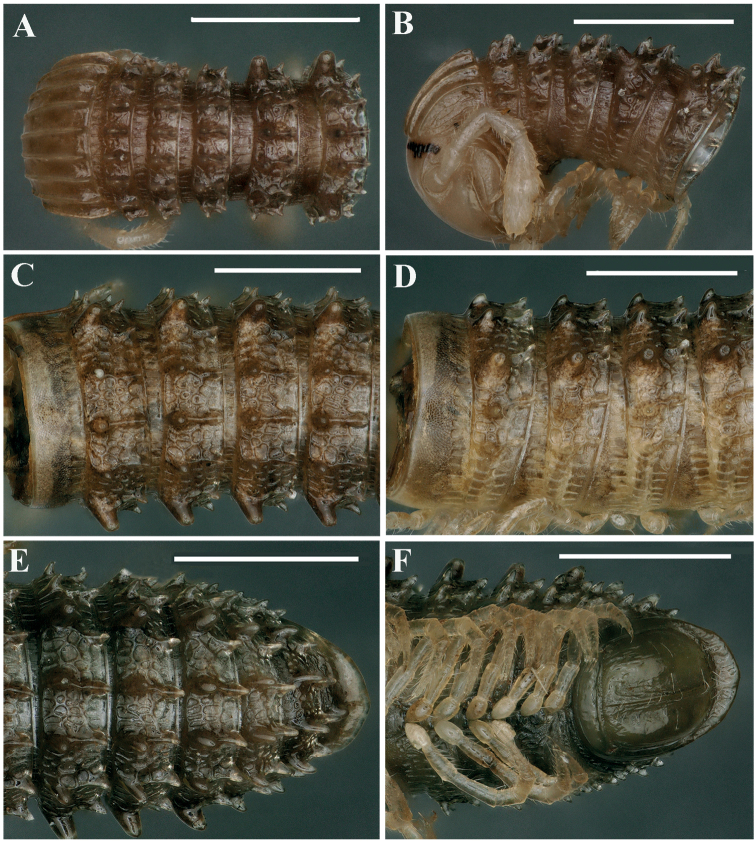
*Glyphiulusxiniudong* Zhao & Liu, sp. nov., ♂ paratype **A, B** anterior body rings, dorsal and lateral views **C, D** mid-body rings, dorsal and lateral views, respectively **E, F** posterior body rings, dorsal and ventral views, respectively. Scale bars: 1 mm.

**Figure 11.**
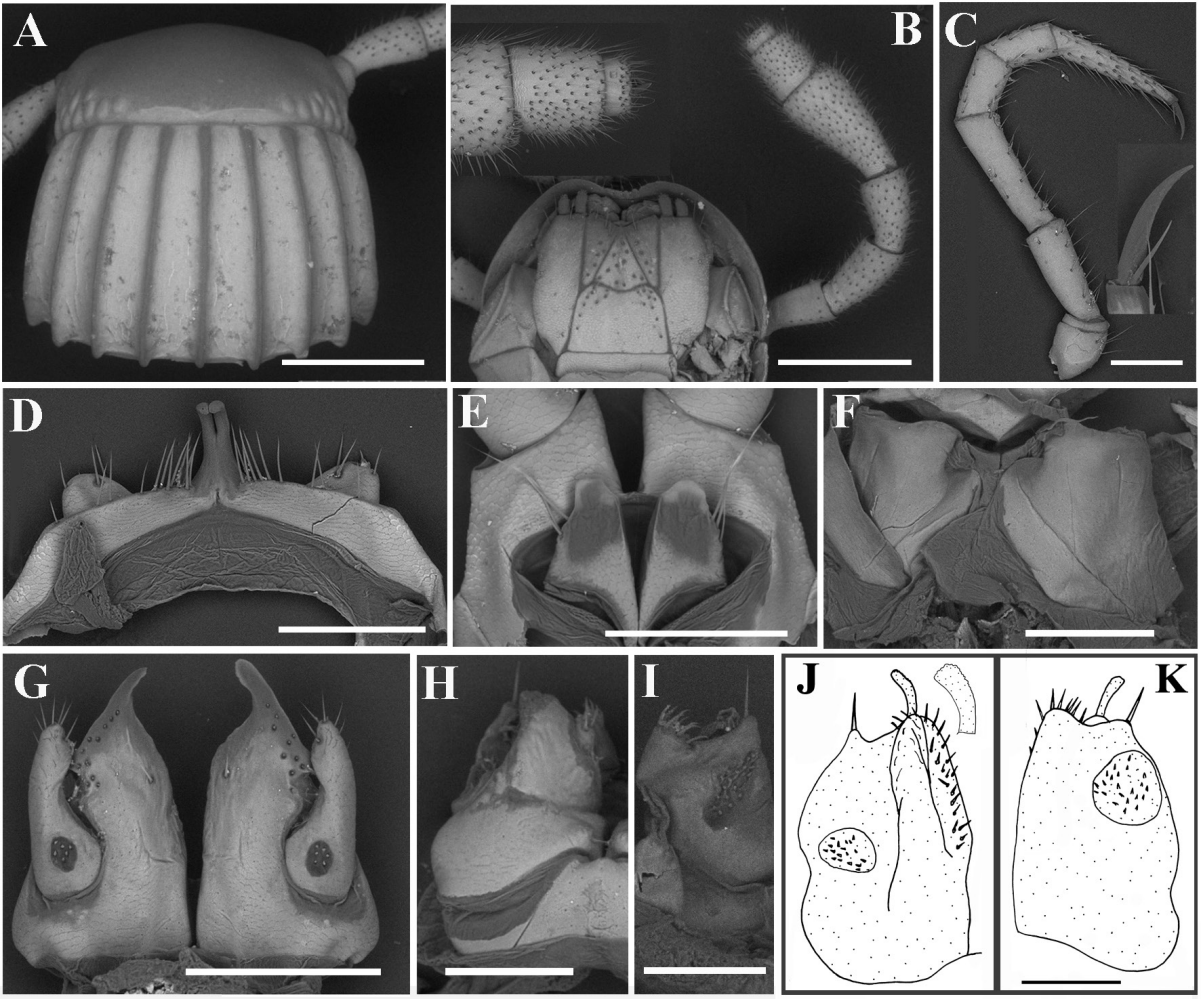
*Glyphiulusxiniudong* Zhao & Liu, sp. nov., ♂ paratype **A** head and collum **B** gnathochilarium and left antenna **C** mid-body leg and claw **D** leg 1, frontal view **E** penes **F** ♀ paratype, vulvae **G** anterior gonopods, caudal view **H, J** posterior gonopod, frontal view **I, K** posterior gonopod, caudal view. Scale bars: 0.5 mm (**A, B**), 0.2 mm (**C–I**), 0.1 mm (**J, K**).

***Head*** surface smooth. Labrum with four teeth anteromedially. Ocellaria blackish, with 9–13 ommatidia arranged in 1–2 irregular linear rows (Figs 10B, 11A). Antennae short, slightly clavate, reaching back to caudal margin of ring 2; in length, antennomeres 5 > 3 > 4 ≈ 2 > 6 > 1 > 7. Antennomeres 5–7 each with a distodorsal field or corolla of bacilliform sensilla (sensory bacilli). Antennomere 7 with four sensory cones (Fig. 11B). Gnathochilarium with a separate promentum, polytrichous on promentum and mentum, lamellae linguales each with 6–7 setae (Fig. 11B). Mandible not dissected.

***Collum***: crests complete and evident; carinotaxy formula I–III+P+M (Figs 10A, 11A). Following metaterga strongly crested; carinotaxy formula 2/2+I/i+3/3 (Fig. 10). Ozoporiferous tubercles very large, coniform. Prozonae delicately alveolate-areolate, fine longitudinal striations in front of stricture. Metazonae with an obvious, corrugate, carved texture (Fig. 10). Rings 2 and 3 with long pleural flaps.

***Epiproct*** simple, caudal edge uneven, with an obvious central tubercle dorsally (Fig. 10E). Paraprocts regularly convex, each with several irregular rows of setae. Hypoproct transversely bean-shaped, with 4+4 small setae (Fig. 10F).

***Legs*** short, about as long as mid-body height; claw with a relatively large accessory spine at base, about half as long as claw (Fig. 11C). ♂ legs 1 very strongly reduced, represented by a sternum showing a pair of small, more slender, fused, paramedian, subunciform prongs directed forward, with about 7–9+7–9 long setae at base; flanked by strongly separated, rudimentary, 1-segmented leg vestiges, with some setae (Fig. 11D). ♂ legs 2 slightly hypertrophied, coxae large; penes rather small, much shorter than coxae, oblong-subtrapeziform, each with three strong setae distolaterally (Fig. 11E). ♂ legs 3 modified through coxae being especially slender and elongate. ♂ femora 6 and 7 normal, neither modifications.

***Anterior gonopods*** (Fig. 11G) with a long subtriangular coxosternum with about 9–11 microsetae near distal margin; apicomesal process of coxite very high, rather narrow and digitiform, modestly curved inwards. Telopodite very large, stout and coiled, 1-segmented, lateral in position, much higher than lateral corner of coxite, with a field of six microspinules at base and 6–7 strong setae apically.

***Posterior gonopods*** (Figs 11H, I) compact. Coxite subquadrate, with a circular field of about 10–15 basolateral microspinules in frontal view; with a field of 19 median microspinules and an apicolateral very strong and long seta in caudal view; apical and mesal parts of coxite with dense, strong and curved setae. Lamelliform lobe membranous, with a rather small, spine-like flagellum.

***Vulvae*** very simple, bare, modestly emarginate medially (Fig. 11F).

###### Remark.

In the absence of direct troglomorphic traits, this species can only be considered as troglophilic at most.

##### ﻿ Glyphiulus scutatus

Taxon classificationAnimaliaSpirostreptidaCambalopsidae

Zhao & Liu sp. nov.

BDC7A359-4DED-5AC6-956E-EF8150CD70DF

http://zoobank.org/9D2EE1BE-88CA-43E0-B7EC-41EBA177FD3E

Figs 5B , 12 , 13

###### Type material.

***Holotype*** ♂ (SCAU WL30), China, Guangxi Zhuang Autonomous Region, Hechi City, Du’an Yao Autonomous County, Napang Dong, 24°08'22"N, 107°51'07"E, 650 m alt., 2015-VII-26, leg. Chen Jujian, Wang Xinhui & Tang Mingruo. ***Paratypes***: 2 ♂, 12 ♀ (SCAU WL30), same data as the holotype. 3 ♂, 4 ♀ (SCAU G72), same County, Cave Bianfu Dong, 24°01'55"N, 108°20'12"E, 550 m alt., 2017-VIII-18, leg. Tian Mingyi, Huang Sunbin, Wang Dianmei & Chen Mengzhen.

###### Etymology.

To emphasise the anterior gonopod showing a scalloped and shield-shaped coxosternum.

###### Diagnosis.

Differs from congeners of the *formosus*-group by both ♂ femora 6 and 7 being slightly inflated and the anterior gonopod without apicomesal process, as well as the posterior gonopod with less than ten microspinules. Based on molecular evidence, *G.scutatus* Zhao & Liu, sp. nov. differs from all other *Glyphiulus* species analysed in a > 15.8% uncorrected p-distance of the COI barcoding gene.

###### Description.

Length of both sexes ca. 25.0–41.0 mm, mid-body rings round in cross-section, their width and height similar, 1.3–1.8 (♂) or 1.8–2.0 mm (♀). Body with 55–68 podous + 1–4 apodous rings + telson. Colouration purple-brownish to dark brownish, legs light brownish to almost transparent (Fig. 5B).

**Figure 12.**
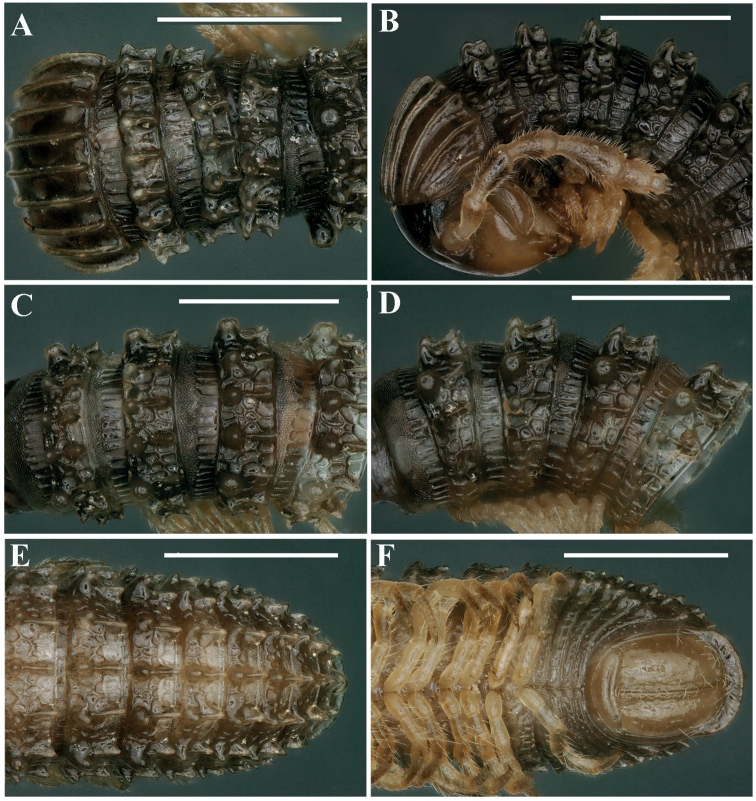
*Glyphiulusscutatus* Zhao & Liu, sp. nov., ♂ paratype from Cave Napang Dong **A, B** anterior body rings, dorsal and lateral views **C, D** mid-body rings, dorsal and lateral views, respectively **E, F** posterior body rings, dorsal and ventral views, respectively. Scale bars: 1 mm.

**Figure 13.**
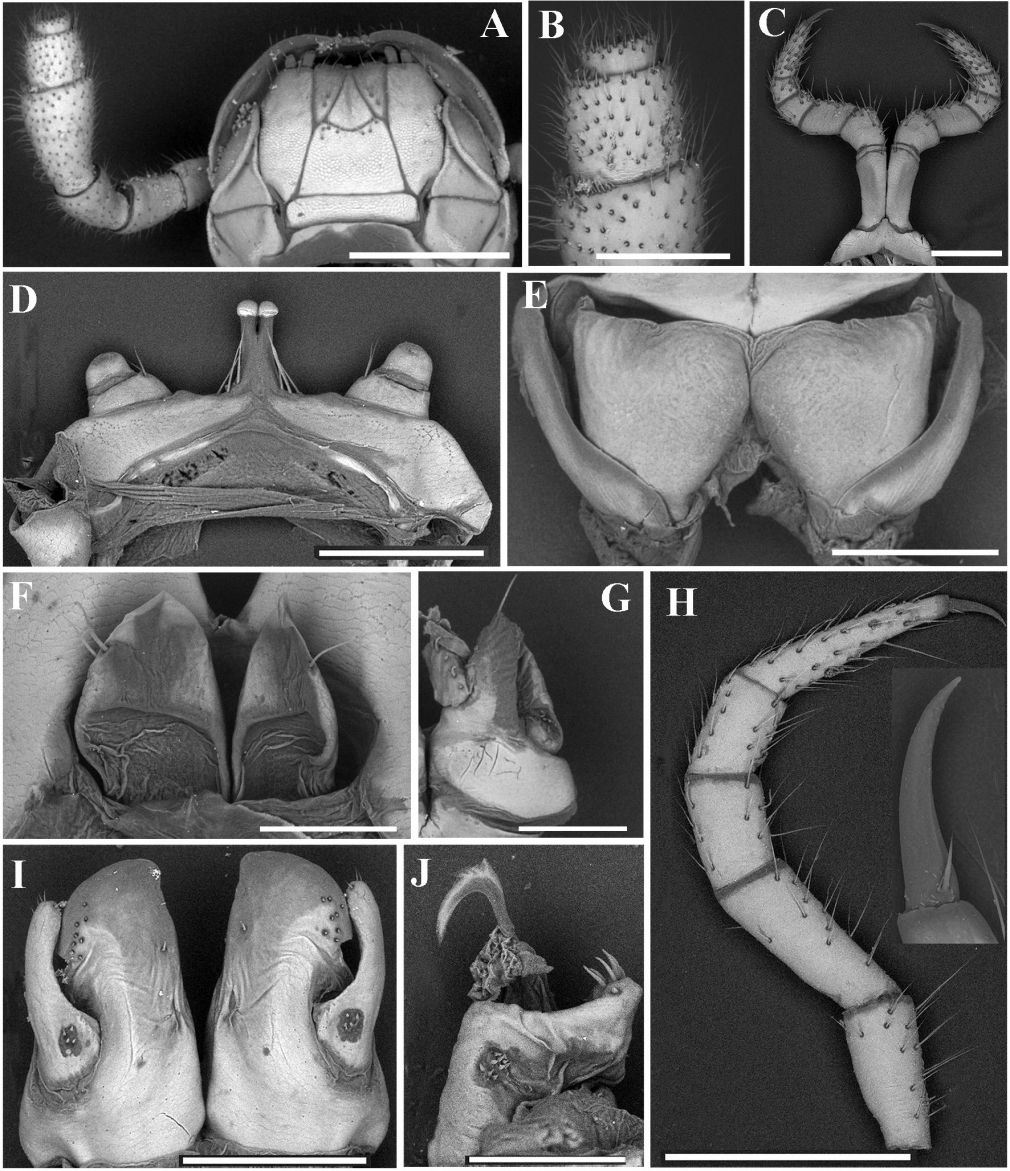
*Glyphiulusscutatus* Zhao & Liu, sp. nov., ♂ paratype from Cave Napang Dong **A** gnathochilarium and right antenna **B** antenna tip **C** leg 3, frontal view **D** leg 1, frontal view **E** ♀ paratype, vulvae **F** penes **G, J** posterior gonopod, frontal and caudal views, respectively **H** mid-body leg and claw **I** anterior gonopods, caudal view. Scale bars: 0.5 mm (**A, H**), 0.2 mm (**B–E**), 0.1 mm (**F, G, I, J**).

***Head*** surface smooth. Labrum with four teeth anteromedially (Fig. 13A). Ocellaria blackish, with 8–12 ommatidia arranged in two irregular linear rows (Fig. 12B). Antennae short, slightly clavate, reaching back to ring 3; in length, antennomeres 5 > 3 > 4 > 2 > 6 > 1 > 7. Antennomeres 5–7 each with a distodorsal field or corolla of bacilliform sensilla (sensory bacilli). Antennomere 7 with four sensory cones (Fig. 13B). Gnathochilarium with a separate promentum, setae variable in number, polytrichous or smooth on promentum and mentum, lamellae linguales each with 4–6 setae (Fig. 13A). Mandible not dissected.

***Collum***: crests complete and evident; carinotaxy formula I–III+P+M (Figs 11A, B). Following metaterga strongly crested and extremely sharp; carinotaxy formula 2/2+I/i+3/3 (Fig. 12). Ozoporiferous tubercles very large, subcylindrical. Prozonae delicately alveolate-areolate, fine longitudinal striations in front of stricture. Metazonae with an obvious, corrugate, carved texture (Fig. 12). Rings 2 and 3 with long pleural flaps.

***Epiproct*** simple, very narrow, caudal edge uneven, with a clear central tubercle dorsally (Fig. 12E). Paraprocts regularly convex, each with several irregular rows of setae. Hypoproct transversely bean-shaped, with 3+3 small setae (Fig. 12F).

***Legs*** slender, about 1.1–1.3 times as long as mid-body height; claw with a small accessory spine at base, about ¼ as long as claw (Fig. 13H). ♂ legs 1 very strongly reduced, represented by a sternum showing a pair of small, fused, paramedian, subunciform prongs directed forward, with 3+3 long setae at base; flanked by strongly separated, rudimentary, 2-segmented leg vestiges, with either a few or no setae (Fig. 13D). ♂ legs 2 slightly hypertrophied, coxae large; penes small, much shorter than coxae, oblong-subtrapeziform, each with 1–4 strong setae distolaterally (Fig. 13F). ♂ legs 3 modified through coxae being especially slender and elongate (Fig. 13C). Both ♂ femora 6 and 7 slightly inflated.

***Anterior gonopods*** (Fig. 13I) with a scalloped and shield-shaped coxosternum with about 6–8 microsetae near lateral corner of coxite. Telopodite very large and coiled, 1-segmented, lateral in position, much higher than lateral corner of coxite, with a field of 5–7 microspinules at base and 4–5 strong setae apically.

***Posterior gonopods*** (Figs 13G, J) compact. Coxite subquadrate, with a field of about 8–10 basolateral microspinules in frontal view and with a field of ten median microspinules in caudal view; apical and mesal parts of coxite with dense, strong and curved setae. Lamelliform lobe membranous, with a short, broad, distally spike-like flagellum.

***Vulvae*** very simple, bare, rather faintly emarginate medially (Fig. 13E).

###### Remark.

In the absence of direct troglomorphic traits, this species can only be considered as troglophilic at most.

##### ﻿ Glyphiulus portaliformis

Taxon classificationAnimaliaSpirostreptidaCambalopsidae

Zhao & Liu sp. nov.

2F6C4A2F-A26D-5C6A-B2EF-C35CFD97C050

http://zoobank.org/5879B2B8-A5DE-46BE-8710-07E3E41BAE46

Figs 5C , 14 , 15

###### Type material.

***Holotype*** ♂ (SCAU WL40), China, Guangxi Zhuang Autonomous Region, Hechi City, Bama County, Cave Baiyan Dong, 24°03'40"N, 107°08'16"E, 400 m alt., 2015-VII-31, leg. Chen Jujian, Wang Xinhui & Tang Mingruo. ***Paratypes***: 7 ♂, 18 ♀ (SCAU WL40), same data as the holotype.

###### Etymology.

To emphasise the coxosternum of the anterior gonopods being portal-shaped.

###### Diagnosis.

Differs from congeners of the *formosus*-group by the epiproct showing a small caudal protrusion and the anterior gonopods being portal-shaped, combined with a foliate flagellum of the posterior gonopod. Based on molecular evidence, *G.portaliformis* Zhao & Liu, sp. nov. Differs from all other *Glyphiulus* species analysed in a > 14.4% uncorrected p-distance of the COI barcoding gene.

###### Description.

Length of both sexes ca. 41.0–56.0 mm, mid-body rings round in cross-section, their width and height similar, 2.2–3.0 mm. Body with 55–67 podous + 1 apodous ring + telson. Colouration brownish, legs almost transparent (Fig. 5C).

**Figure 14.**
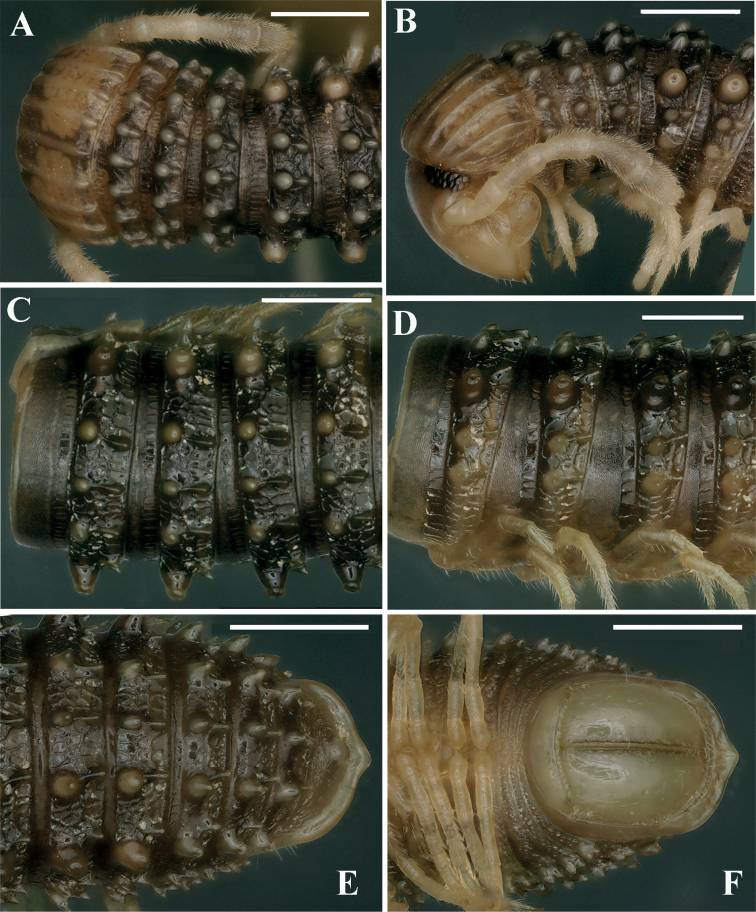
*Glyphiulusportaliformis* Zhao & Liu, sp. nov., ♂ paratype **A, B** anterior body rings, dorsal and lateral views **C, D** mid-body rings, dorsal and lateral views, respectively **E, F** posterior body rings, dorsal and ventral views, respectively. Scale bars: 1 mm.

**Figure 15.**
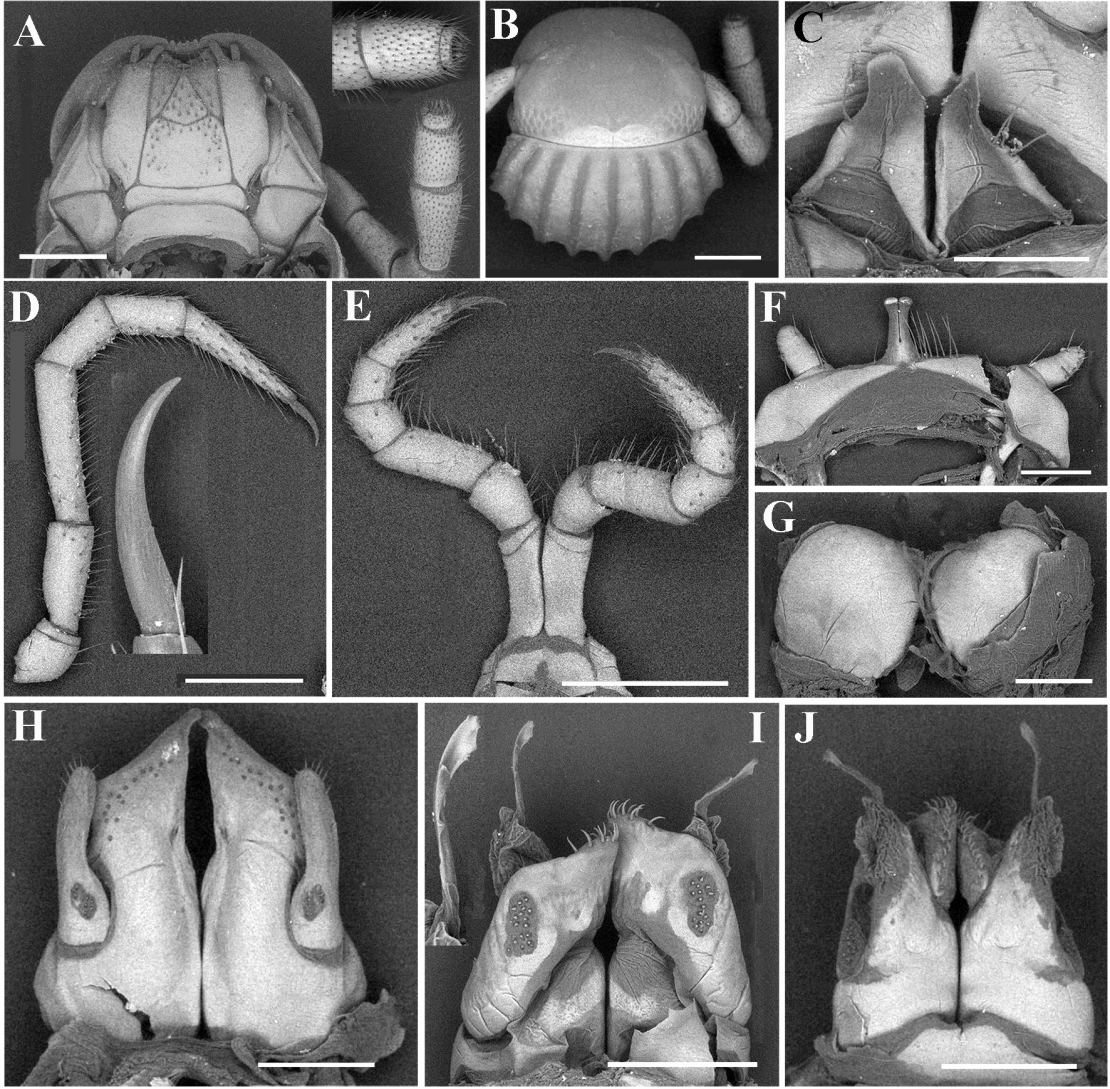
*Glyphiulusportaliformis* Zhao & Liu, sp. nov., ♂ paratype **A** gnathochilarium and left antenna **B** head and collum **C** penes **D** mid-leg and claw **E** leg 3, caudal view **F** leg 1, frontal view **G** ♀ paratype, vulvae **H** anterior gonopods, caudal view **I, J** posterior gonopod, caudal and frontal views, respectively. Scale bars: 0.5 mm (**A, B, D, E**), 0.2 mm (**C, F–J**).

***Head*** surface smooth. Labrum with 4 teeth anteromedially (Fig. 15A). Ocellaria blackish, with 14–17 ommatidia arranged in 2–3 irregular linear rows (Figs 14B, 15B). Antennae short, slightly clavate, reaching back to ring 4; in length, antennomeres 5 > 3 > 2 > 4 > 6 > 1 > 7. Antennomeres 5–7 each with a distodorsal field or corolla of bacilliform sensilla (sensory bacilli). Antennomere 7 with four sensory cones (Fig. 15A). Gnathochilarium with a separate promentum, polytrichous on promentum and mentum, lamellae linguales each with 6–7 setae (Fig. 15A). Mandible not dissected.

***Collum***: crests complete and evident; carinotaxy formula I–III+P+M (Figs 14A, B, 15B). Following metaterga strongly crested; carinotaxy formula 2/2+I/i+3/3 (Fig. 14). Ozoporiferous tubercles very large, coniform. Prozonae delicately alveolate-areolate, fine longitudinal striations in front of stricture. Metazonae with an obvious, corrugate, carved texture (Fig. 14). Rings 2 and 3 with long pleural flaps.

***Epiproct*** simple, caudal edge with a central conical protrusion and dorsally with a strong central rounded tubercle (Fig. 14E). Paraprocts regularly convex, each with several irregular rows of setae. Hypoproct transversely bean-shaped, no setae visible (Fig. 14F).

***Legs*** slender, about 1.1 times as long as mid-body height; claw with a small accessory spine at base, about 1/5 as long as claw (Fig. 15D). ♂ legs 1 very strongly reduced, represented by a sternum showing a pair of small, fused, paramedian, subunciform prongs directed forward, with about ten long setae at base; flanked by strongly separated, rudimentary, 2-segmented leg vestiges, second segment very large, stout, rod-shaped (Fig. 15F). ♂ legs 2 slightly hypertrophied, coxae large; penes small, much shorter than coxae, oblong-subtrapeziform, each with two or three strong setae distolaterally (Fig. 15C). ♂ legs 3 modified through coxae being especially slender and elongate (Fig. 15E). Both ♂ femora 6 and 7 normal, without modifications.

***Anterior gonopods*** (Fig. 15H) very tall, with a portal-shaped coxosternum with about 16–18 microsetae near distal margin; apicomesal process of coxite subtriangular, tip small, slightly curved inwards. Telopodite very large and clavate, 1-segmented, lateral in position, slightly higher than lateral corner of coxite, with a field of nine microspinules at base and six strong setae apically.

***Posterior gonopods*** (Figs 15I, J) compact. Coxite nearly parallelogram-shaped, with an elongated field of about 14 basolateral microspinules in frontal view and with a field of 16 median microspinules in caudal view; apical and mesal parts of coxite with dense, strong and curved setae. Lamelliform lobe membranous, with a relatively long, broad, distally foliate flagellum.

***Vulvae*** very simple, bare, faintly emarginate medially (Fig. 15G).

###### Remark.

In the absence of direct troglomorphic traits, this species can only be considered as troglophilic at most.

##### ﻿ Glyphiulus calceus

Taxon classificationAnimaliaSpirostreptidaCambalopsidae

Jiang, Guo, Chen & Xie, 2018

F987B6B3-E6F7-5F7B-BA07-A6EDF64A9811

Glyphiulus calceus Jiang, Guo, Chen & Xie, 2018: 162.

###### Material examined.

4 ♂, 7 ♀ (SCAU WL37), China, Guangxi Zhuang Autonomous Region, Hechi City, Fengshan County, Zhaiya Town, Cave Jianbang Dong, 24°43'02.96"N, 107°13'11.21"E, 350 m alt., 2015-VIII-4, leg. Chen Jujian, Huang Sunbin & Tang Mingruo.

###### Remarks.

**Figure 16.**
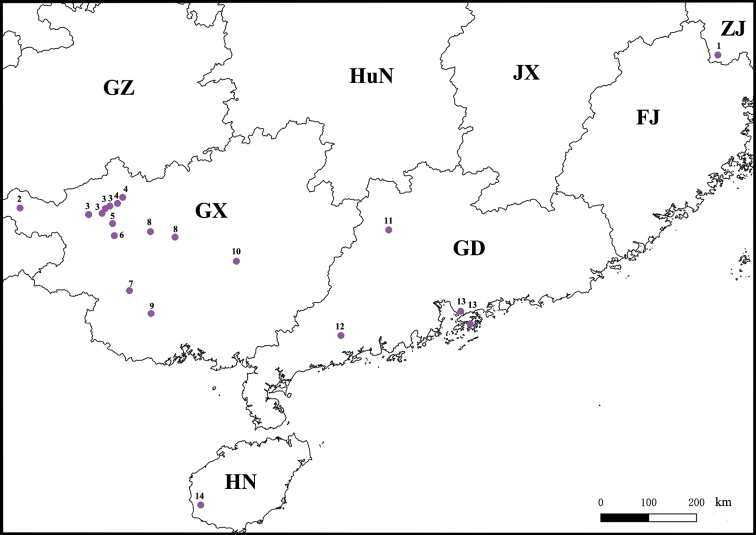
A distribution map of the *Glyphiulusformosus* species group in China. **1***G.recticullus***2***G.foetidus***3***G.impletus***4***G.calceus***5***G.fortis***6***G.portaliformis* Zhao & Liu, sp. nov. **7***G.pulcher***8***G.scutatus* Zhao & Liu, sp. nov. **9***G.echinoides***10***G.xiniudong* Zhao & Liu, sp. nov. **11***G.sinuatoprocessus* Zhao & Liu, sp. nov. **12***G.conuliformis* Zhao & Liu, sp. nov. **13***G.formosus***14***G.hainanensis*. Abbreviations: FJ: Fujian, HN: Hainan, HuN: Hunan, GD: Guangdong, GX: Guangxi, GZ: Guizhou, ZJ: Zhejiang.

This species has been described from the Cave Xianren Dong, Bala Town, Tian’e County, Guangxi, China. The new samples were collected from a cave in the neighbouring county, both being located close geographically (Fig. 16). The above material is in good agreement with the original description by Jiang et al. (2018), while intraspecific p-distance is 2.1%, based on DNA-barcoding. Based on molecular evidence, *G.impletus* differs from all other *Glyphiulus* species analysed from between 11.9% (compared to *G.calceus*) and 23.6% (compared to *G.duangdee*).

##### ﻿ Glyphiulus impletus

Taxon classificationAnimaliaSpirostreptidaCambalopsidae

Jiang, Guo, Chen & Xie, 2018

472267E3-8999-5603-88D0-1EC494A53659

Glyphiulus impletus Jiang, Guo, Chen & Xie, 2018: 171.

###### Material examined.

9 ♂ (SCAU WL38), China, Guangxi Zhuang Autonomous Region, Hechi City, Fengshan County, Jiangzhou Underground Corridor, 24°30'4"N, 106°53'46"E, 850 m alt., 2013-VI-30, leg. Tian Mingyi, Liu Weixin, Lin Wei, Yin Haomin & Huang Sunbin. 4 ♂, 2 ♀ (SCAU WL39), China, Guangxi Zhuang Autonomous Region, Baise City, Linyun County, Cave Fengniu Dong, 24°28'39"N, 106°37'52"E, 700 m alt., 2017-VI-9, leg. Tian Mingyi, Liu Weixin, Wang Xinhui & Tang Mingruo.

###### Remarks.

This species has been found to have a relatively wide distribution in Guangxi, involving the Lingyun, Fengshan, Nandan and Donglan Counties. The new samples add only the records of two cave collections (Fig. 16). The above material is in good agreement with the original description by Jiang et al. (2018), while intraspecific p-distances are 2.4–8.2%, based on DNA-barcoding. Based on molecular evidence, *G.impletus* differs from all other *Glyphiulus* species analysed from between 11.9% (compared to *G.calceus*) and 24.9% (compared to *G.foetidus*).

### ﻿A key to the species of the *Glyphiulusformosus*-group known from China

**Table:** 

1	Anterior gonopod with a scalloped and shield-shaped coxosternum (Fig. 13I)	**2**
–	Anterior gonopod coxosternum not fan-shaped, but with a high apicomesal process of varying shapes (Figs 7H, 9I, 11G, 15H)	**6**
2	♂ leg 1 with a 1-segmented telopodite	***G.pulcher***
–	♂ leg 1 with a 2-segmented telopodite (Fig. 13D)	**3**
3	Collum quadrate; telopodite of ♂ leg 1 with a claw	***G.recticullus***
–	Collum not quadrate; telopodite of ♂ leg 1 without a claw	**4**
4	Neither ♂ femora 6 nor 7 inflated; anterior gonopod coxosternum lower than telopodite	***G* . *echinoides***
–	Both ♂ femora 6 and 7 inflated; anterior gonopod coxosternum higher than telopodite	**5**
5	Anterior gonopod coxosternum with 14–15 microsetae along lateral margin; flagellum of posterior gonopod incurved and sawtooth-shaped at inner margin	***G.formosus***
–	Anterior gonopod coxosternum with 6–8 microsetae near lateral corner; flagellum of posterior gonopod spike-like (Fig. 13J)	***G.scutatus* Zhao & Liu, sp. nov.**
6	Both ♂ femora 6 and 7 inflated, with a small tubercle distoventrally	***G.hainanensis***
–	Both ♂ femora 6 and 7 normal, not modified	**7**
7	Metazonae with an obvious, corrugate, carved texture (Figs 10, 12, 14)	**8**
–	Metazonae relatively smooth, not so markedly carved (Figs 6, 8)	**9**
8	Apicomesal process of anterior gonopod coxite narrow and digitiform (Fig. 11G); posterior gonopod coxite with a long apicolateral seta in caudal view (Fig. 11H, I)	***G.xiniudong* Zhao & Liu, sp. nov.**
–	Apicomesal process of anterior gonopod coxite subtriangular (Fig. 15H); posterior gonopod coxite devoid of a long apicolateral seta (Fig. 15I)	***G.portaliformis* Zhao & Liu, sp. nov.**
9	Metatergal anterior tubercles coniform, unusually sharp (Fig. 8)	***G.conuliformis* Zhao & Liu, sp. nov.**
–	Metatergal anterior tubercles mostly well-rounded	**10**
10	Flagellum of posterior gonopod pectinate distally, with several branches at inner margin	**11**
–	Flagellum of posterior gonopod broad and flat, finely serrate distally at inner margin	**12**
11	Apicomesal process of anterior gonopod coxite digitiform, tip narrow and hook-shaped (Fig. 7H)	***G.sinuatoprocessus* Zhao & Liu, sp. nov.**
–	Apicomesal process of anterior gonopod coxite subtriangular, tip not hook-shaped	***G.foetidus***
12	Posterior gonopod coxite with a long apicolateral seta	***G.calceus***
–	Posterior gonopod coxite without a long apicolateral seta	13
13	Apicomesal process of anterior gonopod coxite very slender, finger-shaped	***G.impletus***
–	Apicomesal process of anterior gonopod coxite rather strong, tip rounded	***G.fortis***

## ﻿Discussion

Morphologically, the Chinese species from the *formosus*-group can presently be considered as well-defined: (1) male leg 1 with a pair of small, fused, paramedian, subunciform prongs directed forward, flanked by strongly separated, rudimentary, 1- or 2-segmented leg vestiges; and (2) collum’s carinotaxy formula I–III+P+M. However, although *G.submediator* Golovatch, Geoffroy, Mauriès & VandenSpiegel, 2011, from Vietnam and *G.striganovae* Golovatch, Geoffroy, Mauriès & VandenSpiegel, 2012, from Borneo, Indonesia, both agree in the above character 1, the carinotaxy formula of the collum in the former species being I–III+4c+5a+pc+ma (Golovatch et al. 2011b), vs. 1c+II+3c+4a+pa+ma in the latter congener (Golovatch et al. 2012). Therefore, we are inclined to treat *G.submediator* as remaining in the original *javanicus*-group. Golovatch et al. (2012) mentioned that *G.striganovae* failed to fit in either the *granulatus*- or the *javanicus*-group. At present, we also have no clear clue for its closer assignment. Nor that *G.striganovae* may not belong to *Glyphiulus* because of its special gonopod structure.

In addition, most species of the Chinese *sinensis*-group show the distal margin of the anterior gonopod coxosternum clearly concave and arcuate centrally and the posterior gonopod sometimes lacks a flagellum. On the contrary, all members of the *formosus*-group either have a scalloped and shield-shaped coxosternum or bear a high apicomesal process of the coxite, while their posterior gonopod always has a flagellum. Based on this, the relationship between the *formosus*-group and the *granulatus*-group may be considered closer from the perspective of the anterior and posterior gonopod structure.

In the single-gene COI phylogenetic tree, the genus *Plusioglyphiulus* may be speculated as being more closely related to *Glyphiulus* than to *Trachyjulus*, because its two species are clustered together with *Glyphiulus* clade BA and clade BB. Golovatch et al. (2011c) also pointed out that some species of *Plusioglyphiulus* appear to be highly peculiar morphologically and there may be transitional groups bridging them with the *javanicus*-group of *Glyphiulus*. Although single-gene COI construction can effectively identify species, a phylogenetic tree, based on the joint construction of multiple genes is deemed to much better resolve the relationship between species (Hebert et al. 2003; Cepeda et al. 2012; Hassan and Hassan 2021).

In our study, *Glyphiulus* proves to be a monophyletic taxon, based on both morphological and molecular phylogenetic evidence. It can presently be divided into three clades, the relationship between them being ((Clade A, Clade B), Clade C), albeit none has gained strong support yet. Therefore, in order to fully verify its reliability, a larger number of samples and a greater amount of information are needed to promote further advance in the study of Cambalopsidae, *Glyphiulus* included.

Most of the *Glyphiulus* species presently known to occur in China appear to largely be confined to the southern parts of the country. Moreover, most of them have been found in caves. Based on the current distribution map (Fig. 16), the *formosus*-group may belong to the South China regionalisation type, mainly covering Guangxi, Guangdong, Hong Kong and Hainan, with solely *G.recticullus* coming from Zhejiang (Zhang and Li 1982). Whether there is indeed an obvious geographic gap/isolation between the *formosus*-group and the other two species groups may become clearer as further species become revealed and/or recorded from intermediate areas.

## Supplementary Material

XML Treatment for Glyphiulus sinuatoprocessus

XML Treatment for Glyphiulus conuliformis

XML Treatment for Glyphiulus xiniudong

XML Treatment for Glyphiulus scutatus

XML Treatment for Glyphiulus portaliformis

XML Treatment for Glyphiulus calceus

XML Treatment for Glyphiulus impletus

## Supplementary materials

Supplementary material 1 Table S1 Data type Uncorrected p-distances Explanation note Uncorrected p-distances for the 657 bp barcoding of the COI gene as between *Glyphiulus* and some related Cambalosidae taxa. https://binary.pensoft.net/file/705267This dataset is made available under the Open Database License (http://opendatacommons.org/licenses/odbl/1.0/). The Open Database License (ODbL) is a license agreement intended to allow users to freely share, modify, and use this Dataset while maintaining this same freedom for others, provided that the original source and author(s) are credited.Yi Zhao, Wanru Guo, Sergei Golovatch, Weixin Liu
